# Acute Effect of Whole-Body Vibration on Trunk Endurance and Balance in Obese Female Students: Randomized Controlled Trial

**DOI:** 10.3390/medicina60081316

**Published:** 2024-08-14

**Authors:** Nesma M. Allam, Raghad Miah Alenzi, Lashin Saad Ali, Shaden Mohammed Al Muteb, Sara Abdulkarim Aljabar, Hind Fahad Altuwayrib, Renad Fayez Al-Mashaiti, Welf Fahad Albarak, Dalia Mahmoud Abdelmonem Elsherbini, Rasha Hamed Al-Serwi, Ateya Megahed Ibrahim, Mamdouh Eldesoqui, Mohamed El-Sherbiny

**Affiliations:** 1 Department of Physical Therapy and Health Rehabilitation, College of Applied Medical Sciences, Jouf University, P.O. Box 2014, Sakaka 72388, Saudi Arabia; nmallam@ju.edu.sa (N.M.A.); raghad1832002@hotmail.com (R.M.A.); shadenalmoteb@gmail.com (S.M.A.M.); xsara67x@gmail.com (S.A.A.); hind_121@outlook.sa (H.F.A.); srrty811@icloud.com (R.F.A.-M.); wellf.fahad.1423@gmail.com (W.F.A.); 2 Department of Physical Therapy for Surgery, Faculty of Physical Therapy, Cairo University, Giza 12613, Egypt; 3 Department of Basic Medical Sciences, Faculty of Dentistry, Al-Ahliyya Amman University, Amman P.O. Box 19328, Jordan; l.lashin@ammanu.edu.jo; 4 Department of Medical Physiology, Faculty of Medicine, Mansoura University, Mansoura 35516, Egypt; 5 Department of Clinical Laboratory Sciences, College of Applied Medical Sciences, Jouf University, P.O. Box 2014, Sakaka 72388, Saudi Arabia; 6 Department of Basic Dental Sciences, College of Dentistry, Princess Nourah bint Abdulrahman University, P.O. Box 84428, Riyadh 11671, Saudi Arabia; rhalserwi@pnu.edu.sa; 7 College of Nursing, Prince Sattam bin Abdulaziz University, Al-Kharj 11942, Saudi Arabia; a.eleglany@psau.edu.sa; 8 Department of Family and Community Health Nursing, Faculty of Nursing, Port Said University, Port Said 42511, Egypt; 9 Department of Basic Medical Sciences, College of Medicine, AlMaarefa Universit, P.O. Box 71666, Riyadh 11597, Saudi Arabia; mamrah@um.edu.sa (M.E.); msharbini@um.edu.sa (M.E.-S.); 10 Department of Anatomy, Faculty of Medicine, Mansoura University, Mansoura 35516, Egypt

**Keywords:** whole body vibration, trunk endurance, balance, obese females

## Abstract

*Background and Objectives:* Compared to other subjects, obese people have inferior trunk muscle endurance and balance. A modern method of neuro-muscular training called whole body vibration (WBV) may improve trunk muscle endurance and balance. This study evaluates the impact of a 4-week WBV program on trunk endurance and balance in obese female students. *Materials and Methods:* Sixty participants from 18 to 25 years of age and with BMI values ≥ 30 were randomly distributed into two equal groups: Group A (WBV group), who received 4 min of WBV, and Group B (sham WBV group), who received WBV with a turn-off device. The training was conducted two days/week for six weeks. Trunk endurance was evaluated using the Sorensen Test (ST) and Trunk Flexor Endurance Test (TFET). The Single-Leg Test (SLT) was used to assess static balance, while the Biodex Stability System measured dynamic balance. *Results*: The current study demonstrated no significant differences (*p* > 0.05) in pre-treatment variables between Groups A and B. Post-treatment, Group A showed a significantly higher duration of the Sorensen test, TFET and SLS than Group B (*p* < 0.001). Moreover, Group A showed significantly lower dynamic balance (*p* < 0.001) than Group B. *Conclusions:* WBV has a short-term effect on trunk endurance and balance in obese female students. WBV can be added to the rehabilitation program for obese subjects with deficits in trunk endurance and balance.

## 1. Introduction

Obesity is a medical disorder characterised by extra fat accumulation in the body, negatively impacting health [1]. Insulin resistance and hypertriglyceridemia in obese subjects cause elevated blood sugar, higher fasting glucose levels, inflammatory conditions and abdominal fatty tissue [2]. Obesity is also linked to physiological changes in the muscles, such as a reduction in blood flow, which limits the delivery of oxygen and energy sources. These physiological alterations may hasten the onset of muscle fatigue when performing continuous contractions [3]. Adolescents with obesity had reduced levels of regular physical activity, which is typically expected to decrease their muscular activation capability [4].

There is quantitative proof that obesity negatively impacts balance because it alters the body’s geometry by adding passive weight to various body areas, affecting the biomechanics of daily activities, which can lead to functional restrictions and possibly increase the risk of injury [5]. Also, it may play a significant role in the incidence of falls, which helps in clarifying why obese individuals seem to be more vulnerable than those of normal weight to everyday postural stresses and disruptions [6].

Kibler describes core/trunk muscles as having “the capability to enable maximum generation, transfer, and management of motion and force to the distal part as well as to regulate the orientation and movement of the trunk relative to the pelvis” [7]. These muscles include the hip and lumbopelvic muscles. The ability to labour at a moderately intense level for a lengthy amount of time while still preserving the overall function of the muscular system is known as trunk endurance [8]. The fundamental job of trunk muscles is to maintain the spine’s stability, which is crucial for all daily activities, together with neuro-muscular control, ligaments and osseous support [9].

Previous studies explained why obesity negatively influences balance by two theories: First, the plantar sensitivity is decreased due to the plantar mechanoreceptors being hyper-activated to sustain the heavy mass under constant pressure. Second, they have a higher mechanical demand because their entire body’s centre of mass is located farther from the axis of rotation, which results in a larger gravitational torque [10,11].

Whole body vibration (WBV) is a type of neuromuscular facilitation used to improve overall wellness, performance and rehabilitation. It has become an effective replacement technique that requires less time to apply and produces more immediate improvements in strength and balance [12]. In WBV training, the platform generates vertical sinusoidal vibration. These mechanical inputs enter the body and activate sensory receptors, especially the muscle spindles [13], improve recruitment thresholds for motor units and synchronisation and activate both synergists and antagonists [14]. Platform movements can generate perturbations that enhance balance by activating sensory receptors in muscles, joints and ligaments. These receptors can then trigger the reflex circuit. Therefore, adapting the neuromuscular system produced by WBV can cause physiological changes like those in traditional strength and endurance training [15].

The training protocol may have an impact on the efficacy of WBV regarding the parameters of vibration, including the frequency, amplitude and application technique, in addition to the intervention (type of training, volume and intensity), mainly when applied over extended periods [16]. Several reviews have shown that the WBV protocols lack sufficient rigour and that various processes, participants, intervention features and vibration platforms affect the study’s conclusions and comparability [17,18]. WBV has been established in rehabilitation programs to improve trunk endurance in sub-populations such as healthy young adults [19] and older populations [20]. In addition, other studies evaluated the impact of WBV on balance in burn patients [21], the elderly [20] or those suffering from neurological conditions, including Parkinson’s [22], multiple sclerosis [23] or stroke [24]. Up until now, the benefits of WBV training on trunk endurance and balance in obese females have rarely been studied. Muscular endurance is essential for performing recurrent tasks of daily living, including ascending stairs or walking, so measuring it is even more crucial when evaluating physical performance [25]. In addition, body imbalance leads to difficulty in performing several daily activities. Consequently, it would seem that obesity is related to a higher risk of falling [26]. Thus, this study aims to determine if WBV training causes an acute enhancement in trunk endurance and balance in obese female students compared to a sham control conducted without vibration.

## 2. Materials and Methods

### 2.1. Study Design

This was a randomised, double-blinded, parallel-group, prospective, sham-controlled trial conducted from December 2023 to May 2024 at the College of Applied Medical Sciences, Jouf University. This study was approved by the Ethics Committee of Qurayyat Health Affairs (IRB No. 2023-125). It was registered at ClinicalTrials.gov (NCT06202365) following the principles of the Declaration of Helsinki. The study’s objectives, methods and steps were first explained to the participants, and those selected were asked to sign an informed consent before participation. The present study followed the CONSORT guidelines.

### 2.2. Subjects

Sixty female participants were initially selected in this study. The inclusion criteria were BMI values ≥ 30 and ages from 18 to 25 years old. All those with pregnancy, musculoskeletal disorders (bone fracture in the last year, history of spinal surgery, metal plates in the body, acute or chronic low back pain or ligament sprain), implanted cardiac pacemakers, menstrual irregularities, drugs that may have an impact on the musculoskeletal system, a history of engaging in frequent exercise > three times/week for the last six months, tumours or chronic diseases such as cardiovascular, respiratory, abdominal, urinary, gynaecological and neurological diseases, hypertension or diabetes mellitus were excluded. The participants were distributed into two equal groups: Group A (WBV group) received WBV for 4 min two times a week for six weeks, and Group B (Sham WBV group) received WBV with a turn-off device two sessions a week for six weeks. The assessment was conducted before the intervention and six weeks after the training for both groups.

### 2.3. Sample Size

Based on the prior research by Abbasi et al. [27], the sample size was calculated with α = 0.05. It was determined that 26 participants in each group, or a total of 52 participants, would have a power of 80% and a 0.70 effect size based on the primary outcome, the scores of Sorenson’s test. Each group consisted of 30 patients, allowing for a 15% dropout rate.

### 2.4. Randomisation

Block randomisation was used to assign the participants to the two groups, WBV and control (Sham WBV group), by an independent physical therapist who was not engaged in evaluation or treatment procedures using a computer-based program at http://www.randomization.com/, accessed on 31 December 2023. With an allocation ratio of 1:1, the participants were randomized in blocks of four to minimise the bias and variation in both groups. Not all caregivers and other study personnel were aware of the randomisation process.

### 2.5. Procedures

Approximately one week before gathering the data, all participants were given familiarisation sessions for the WBV and assessments. Before the assessments, each participant warmed up using the same 2 min routine: static stretching for lower limb muscles and trunk rotation exercises. Each stretch exercise was repeated three times on each side for 10 s per repetition. Two sets of fifteen twists on each side were completed as a warm-up for trunk rotation [28]. None of the participants conducted any exercise besides the training program included in the study. In order to ensure that there was no weight loss, participants’ weight was checked two times per week in combination with the WBV training.

#### 2.5.1. Intervention

##### Study Group

After finishing the first evaluation, each participant was instructed to maintain standing on the WBV device (Power Plate ^®^ pro7HC™, Chicago, IL, USA) with their feet parallel at shoulder width apart, grasping the machine’s handlebars and conducting six training exercises (Table 1). The exercises were performed twice a week for six weeks, lasting four minutes per session; the frequency was set at 15–30 Hz, the peak-to-peak amplitude was set at 2 mm and there was a 30 s break between exercises. The frequency of vibration rose throughout the vibration at intervals of one minute, as follows: 1st minute (15 Hz), 2nd minute (20 Hz), 3rd minute (25 Hz) and 4th minute (30 Hz) [12,28,29]. According to other studies [29,30,31], enhancing muscle performance and balance is most effectively accomplished by low amplitudes and frequencies.

While exercising, the participants wore the same gymnastics shoes, distributing their weight equally across both feet on the plate to prevent contusions. In order to decrease resonance and soften the vibration waves transmitted to the body, all participants were instructed to contract their lower limb muscles throughout the vibration. They could also place additional weight on their forefoot [15]. Since evidence-based WBV programs exist, the training program used in this study was selected using comparable protocols shown to enhance performance [32]. The purpose of selecting this training program was to ensure that the body was subjected to a balanced, multidirectional vibration loading and to reduce the monotony of standing on the platform [29]. In addition, rehabilitation programs for obese subjects should include dynamic exercises aimed at improving postural balance training to enhance security and maintain autonomy while preventing falls and injuries [33]. A licensed physical therapist monitored the WBV training.

##### Control Group

The participants in the control group carried out similar exercises and wore the same gymnastics shoes throughout the training. However, the WBV device did not vibrate for the control group.

#### 2.5.2. Outcome Measures

The demographic data for all participants were recorded before starting the study. The first author, who was not included in the randomisation, evaluated all outcome measures before the intervention and at the sixth week after the treatment program’s end. The Sorensen test was the primary outcome; the secondary outcomes were the trunk flexor endurance test, dynamic balance and single-leg stance test.

##### Sorensen Test

The Sorensen test was used to assess the trunk extensor endurance. This test’s Intraclass Correlation Coefficient (ICC) was equal to 0.81, indicating a very high reliability level [34]. The participants were asked to extend their knee and hip joints while lying prone on the bed, keeping the level of the pelvis on the plinth’s edge. The hips, thighs and knees were stabilised in the bed using three straps. The upper trunk first rested on a table at the plinth’s level. The participant was then instructed to actively contract their trunk extensors to maintain their upper trunk at the same level as the remaining body parts and to hold this position for as long as possible. A stopwatch recorded the time for which the subject stayed in this position. The test would end if the subject could not keep the trunk angle horizontal and dropped >10°. After three repetitions, the average time was determined [35].

##### Trunk Flexor Endurance Test (TFET)

The TFET was used to assess trunk flexor endurance. It was proven to have a high interrater reliability, with an ICC of 0.97 [34]. The participants were instructed to keep a position of crook lying with their upper trunk remaining in a 60° flexion using a pivoting, movable plinth. The legs were strapped to the plinth, and the hands were folded across the chest. The participant was instructed to stay in that position as long as possible while the plinth’s upper support was removed from her back. When the subject’s upper body angle dropped by >5° below the standardised testing angle, the test was ended, and the amount of elapsed time was noted. Following the completion of three trials, the average time was determined [27].

##### Single-Leg Stance Test (SLS)

The SLS test is widely used for assessing balance ability and is a strong indicator of falling. With closed eyes, the ICC 0.998 indicates excellent inter-rater reliability [36]. The participants crossed their arms over their chest, maintained the standing position on the dominant leg, raised the non-dominant limb with closed eyes about 5 cm above the medial malleolus of the dominant leg and held that position for as long as possible [37].

A digital stopwatch was utilised for recording time. After the occurrence of any of the following, the test was stopped: (1) the participant moved the raised limb toward or away from the stance limb or contacted the floor, (2) the participant moved the crossed arms, (3) the participant shifted the weight from the foot on the floor to enhance balance, (4) the participant opened her eyes or (5) after 45 s maximally. After three trials, the analysis included the trial with the best score [36].

##### Dynamic Balance

Dynamic balance was evaluated using a postural sway device (Biodex Stability System, New York, NY, USA). The Anterior–Posterior Stability Index (APSI) and Medial–Lateral Stability Index (MLSI) represent the movement of the centre of the platform in the sagittal and frontal planes, respectively. The Overall Stability Index (OSI) reflects the total variance in the movement of the platform centre (APSI and MLSI). These indexes represent the standard deviations evaluating the degree of sway around the central zero point of the platform in which high scores reflect poor balance. The indexes were measured in degrees [38].

The participants were asked to stand comfortably on both feet without shoes on the platform, opening their eyes and having their arms rest next to their trunks. They were instructed to keep this position until finishing the test. There was a 30° angle between the feet and around 8 cm between the heels. Following the test’s directions, the participants performed a trial with uncollected data to see if the participants could comprehend and adhere to the test directions. Every test was carried out for five repetitions, each lasting 30 s, with a rest period of 10 s. Then, each test’s mean scores were determined [8] with acceptable reliability, an ICC of 0.69 [39].

### 2.6. Statistical Analysis

The data were analysed using GraphPad Prism version 9, presented as the mean ± SD. A t-test was used to identify the differences in demographic data between the WBV and Sham WBV groups. The outcome measures between both groups were evaluated using a two-way repeated-measures ANOVA. A percentage was used to represent the qualitative data. The chi-square test was used to compare the proportions. The effect size between-group effect was determined by using a partial eta square. When *p* < 0.05, significance was taken into account. Within each group, the differences in the outcomes were tested using the paired *t*-test. After the data were standardised, any outliers were removed. The associations between the Sorensen test and OSI, the Sorensen test and SLS test and the TFET and OSI pre-intervention and post-intervention in both groups were examined using a linear regression model with a generalised estimating equations (GEE) adjustment. The variance’s normality and homogeneity were statistically assessed before applying the parametric assumption.

## 3. Results

**Figure 1:**
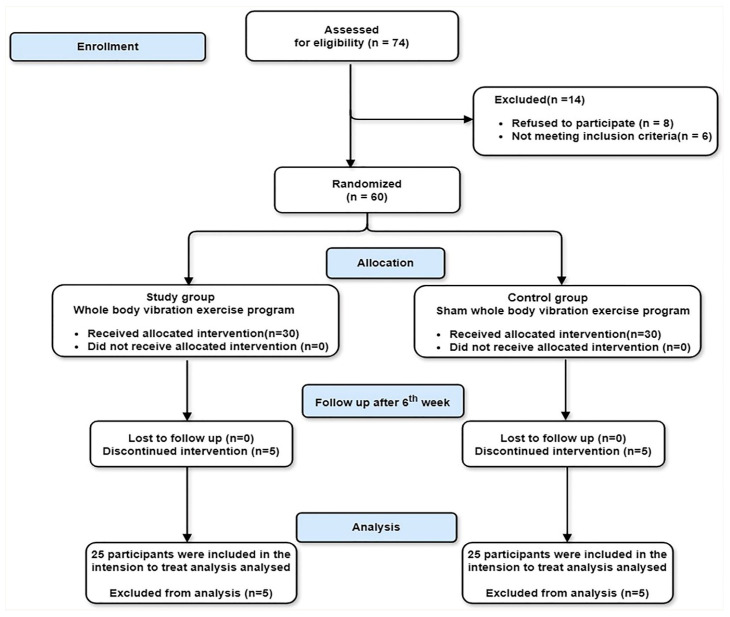
Flowchart for participant recruitment and allocation.

Seventy-four participants were selected for participation in the present study. Fourteen participants were excluded (eight refused to participate, and six did not match the inclusion criteria). Sixty participants were randomly allocated into Group A and Group B. Five participants from each group did not complete the intervention due to heavy schedules for these students. The multiple imputation approach was intended to be utilised in the study to account for the missing data, as shown in Figure 1. Three patients reported side effects after the first session of WBV. Two subjects had leg itching, and one participant reported a headache. After the completion of the trial, all participants showed the same level of adherence to the WBV training in both groups.

### 3.1. Subject Characteristics

Table 2 illustrates the characteristics of the participants of Group A and Group B. No significant difference was reported in the mean age, height, weight or BMI (*p >* 0.05) between both groups.

### 3.2. Clinical Measures

**Figure 2:**
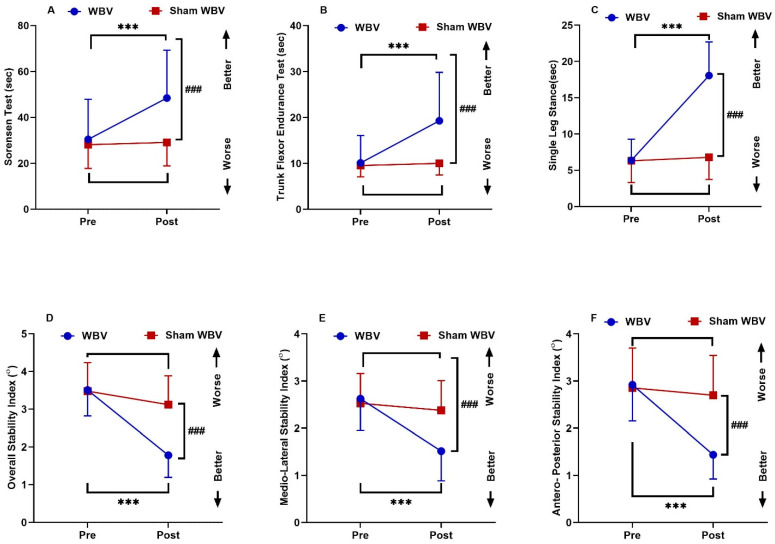
Interactions between group and time for (**A**) Sorensen test (s), (**B**) TFET (s), (**C**) SLST (s), (**D**) OSI, (**E**) MLSI and (**F**) APSI. Data are expressed as the mean ± SD. *** *p* < 0.001 within WBV group pre- vs. post-intervention, ### *p* < 0.001 of WBV vs. Sham WBV group post-intervention.

Post-treatment, the values of the Sorensen test, TFET and SLS increased significantly (*p* < 0.001). At the same time, OSI, MLSI and APSI decreased significantly compared to those before treatment in Group A (*p* < 0.001). In Group B, there was a non-significant difference between the Sorensen test, TFET, SLS, OSI, MLSI and APSI (*p >* 0.05). There were no significant differences for all variables between both groups pre-treatment (*p >* 0.05). While a significant increase was noted in the Sorensen test, TFET and SLS in Group A when compared with those of Group B (*p* < 0.001), and there was a significant decrease in the mean values of OSI, MLSI and APSI in Group A (*p* < 0.001) after the comparison between both groups post-treatment (Table 3 and Figure 2).

### 3.3. Correlation between Sorensen Test and OSI, Sorensen Test and SLS Test and TFET and OSI

**Figure 3:**
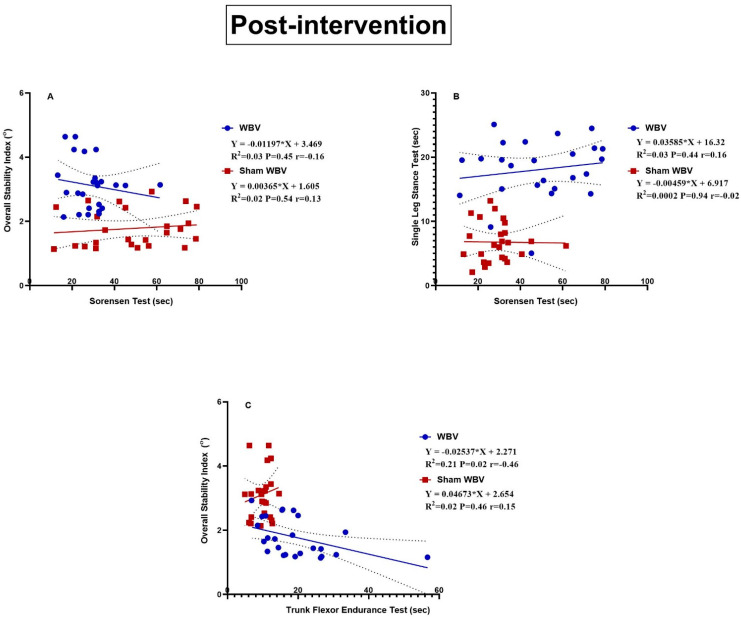
Linear regression of the following parameters post-intervention: (**A**) Sorensen test and OSI, (**B**) Sorensen test and SLST and (**C**) TFET and OSI.

Figure 3A demonstrates a weak correlation between the Sorensen test and OSI, which is positive in the Sham WBV group (r = 0.13) but negative in the WBV group (r = −0.16). The linear regression reported statistical insignificance regarding this. In the Sham WBV group, the increased endurance of trunk extensors explains 2% of the increase in OSI. However, the increased endurance of trunk extensors in the WBV group explains a 3% decrease in OSI. (B) demonstrates a weak positive correlation between the Sorensen and SLS tests in the Sham WBV groups (r = 0.02) and WBV groups (r = 0.16). There were statistically non-significant results for the linear regression. The increased endurance of trunk extensors explains (0.02% and 3%) the increase in the static balance assessed by the SLS test in the Sham WBV and WBV groups, respectively. (C) demonstrates a weak positive correlation between the trunk flexor endurance test and OSI in the Sham WBV group (r = 0.15) but a moderate negative correlation in the WBV group (r = −0.46). The linear regression demonstrated statistical insignificance regarding this in the Sham WBV group but statistical significance in the WBV group (*p* = 0.02). In the Sham WBV group, an increase in the trunk flexor endurance test explains a 2% increase in OSI. However, an increased trunk flexor endurance test in the WBV group explains a 21% decrease in OSI.

## 4. Discussion

Excess weight has been shown to put stress on the soft tissues, joints and bones, leading to impairment of the function of the musculoskeletal system, like aberrant body mechanics [40]. It also leads to different problems, including deficits in balance, locomotion, strength, sensory function, endurance and neuromuscular function, which are significant risk factors for falls. It has been noted that females with obesity have a worse balance than males with obesity and, therefore, are more likely to fall [41]. This highlights the critical role of exercise regimens in improving this population’s trunk endurance and balance. Thus, the current study aimed to determine if obese females might improve their trunk endurance and balance after a 6-week WBV training program.

The current study’s frequency and amplitude were chosen based on prior studies [42,43], which found that the combination of low frequencies (30 Hz) with low amplitudes (2–4 mm) or of high frequencies (50 Hz) with high amplitudes (4–6 mm) produced the highest impact of acute WBV on neuromuscular functioning [42]. Cormie et al. [43] demonstrated that low-frequency (30 Hz) and low-amplitude (2.5 mm) WBV training immediately enhances the functional performance of the muscles.

The current study’s findings revealed a significant increase in the Sorensen test and the TFET in the WBV group compared with those of the Sham WBV group (*p* < 0.001) after comparing the two groups following treatment. The underlying mechanisms for the favourable impact of WBV on trunk endurance might be due to the neuromuscular cause, as it has been reported that WBV has been shown to trigger the tonic vibratory reflex (TVR), which generates the contraction of muscles. The muscle spindles and polysynaptic pathways would be activated via TVR through Ia afferent fibres, thus boosting motor unit recruitment [28]. Moreover, the repeated eccentric-concentric contraction resulting from the WBV exposure in the muscles raises muscle metabolism. Hence, these factors may impact muscular characteristics, such as endurance and strength [44].

The increased activation of fast twitch muscle fibres could explain increased endurance; hence, WBV may alter the neuromuscular activation of core muscles [27]. In addition, WBV boosted prefrontal cortex cerebral oxygenation responses. Higher levels of neural activation are directly correlated with increased cerebral oxygenation and blood flow [45]. Based on this information, it can be assumed that both high cortical adaptations and alterations in the motor unit influenced the current study’s findings.

Our study, consistent with previous studies, revealed that WBV improves trunk endurance in different populations [27,28,46,47]. Ye et al. [28] found that following WBV training at 25 Hz, trunk extensor endurance improved compared to training at 40 Hz. Muscle fatigue is a possible explanation for the reduced effect of high frequency [35]. So, low-frequency vibration was more effective for improving trunk endurance. Similarly, Beglari Neshat et al. [46] showed a significant difference in the trunk extensor endurance test 30 min after WBV in healthy young females.

Additionally, Wirth et al. [47] reported a mild to moderate improvement in muscle activation due to WBV during static exercises for trunk muscles in young, healthy adults. In addition to the WBV’s defining characteristics, such as frequency and amplitude, this effect appears to depend on the distance from the corresponding muscle to the vibration platform and the degree to which the exercise position challenges body balance. Furthermore, Abbasi et al. [27] confirmed that a 6-week WBV training program can effectively impact the core muscles’ strength and endurance in patients with multiple sclerosis without causing adverse side effects. These findings suggested that adaptive modifications after vibration training are not confined to the lower limb; WBV affects muscle activation throughout the body, including the core muscles.

In contrast to our findings, a study conducted by Osawa and Oguma [48] proved that 12 weeks of WBV exercises showed no noticeable changes in muscle endurance in healthy young adults. These contradictory results may be attributed to the poor level of physical fitness or variations in maximal muscular strength at baseline. In addition, each participant had a varied number of training sessions at each vibration frequency, and the control group did not conduct WBV training.

In terms of static and dynamic balance, the results demonstrate a significant increase in SLS in group A when compared with that of group B (*p* < 0.001) and a significant decrease in the mean values of OSI, MLSI and APSI in group A (*p* < 0.001) after a comparison between both groups post-treatment. The mechanism by which WBV affects balance might be because WBV activates the skin’s mechanoreceptors and the muscle joints surrounding the foot and ankle joints. In addition, WBV causes many alterations in the muscles and nervous system. Vibration (1–30 Hz) given to the whole body has been revealed to produce a response known as TVR, which can increase motor unit recruitment, enhance contractile characteristics and muscle strength and help modify proprioception, which improves balance. A postural control mechanism may also play a role in enhancing balance [12,31].

In addition, the lower extremity flexor and extensor muscles co-activate more acutely due to the activation of the neuromuscular system following WBV. This co-activation is thought to have positively influenced postural control techniques throughout WBV [49]. Furthermore, a correlation was observed between the enhancement of neuromuscular control, ankle improvements [50] and knee joint stabilisation [51] following WBV training. This relationship suggests that joint stability may improve balance capability [51].

According to these results, previous studies reported that WBV can enhance balance [31,52,53,54,55,56,57,58,59,60]. Torvinen et al. [31] proved that one session of WBV for 4 min produced a significant, immediate improvement in balance. It was proven that the immediate impact of a short period of vibration improved physical performance. Ritzmann et al. [52] also revealed that WBV improves balance control in healthy, trained individuals. Following a 4-week WBV training program, there was a considerable reduction in the medio-lateral and anterior-posterior displacement of COP. At the same time, the control group did not experience any significant changes.

Similarly, Maeda et al. [53] showed a more significant improvement in the balance than that of the non-WBV group in healthy adults. Schlee et al. [54] observed balance improvement following a 4 min WBV session, which may be attributed to neuromuscular strategies instead of enhanced foot sensitivity. Moreover, Runge et al. [55] proved that WBV training helps elderly patients with their postural control, while van Nes et al. [56] observed that WBV training increased the weight-shifting speed of stroke patients with unilateral disability. Consequently, it seems that WBV benefits various age groups and demographics.

Similar results were found in patients suffering from diabetic peripheral neuropathy (DPN); Waheed et al. [57] reported that functional balance improved with WBV combined with balance exercise instead of balance exercises alone. Kordi Yoosefinejad et al. [58] determined that WBV at a 30 Hz frequency and 2 mm amplitude enhances balance. Another trial demonstrated that WBV can positively affect the balance of patients’ who had DPN [59]. The same results were found in elderly DPN patients who enhanced dynamic and static balance following WBV training in combination with balance exercise [60].

However, the current findings are inconsistent with those of Torvinen et al. [35], who found that young, healthy volunteers’ balance was unaffected by a single, 4 min WBV session. They also reported that despite the beneficial effects on muscle strength, its effectiveness regarding balance remains unknown. They suggested that the vibration stimulus was sufficiently long to influence the thigh and hip muscles’ EMG characteristics but remained too brief to significantly alter the balance or the assessments of muscle performance. Additionally, Moffa et al. [61] reported that WBV does not appear to cause a substantial acute disturbance or adjustment of posture in athletes who are sighted or blind. The cause of these findings might be attributed to the different frequencies of vibration applied by the previous study [61], the alteration in the levels of proprioception in the participants and the short duration of application (only one session of WBV; five sets with 1 min/set).

Regarding the Sorensen test and OSI correlation, the present study found a weak positive correlation in Group B (r = 0.13) but a negative correlation in Group A (r = −0.16). Moreover, a weak positive correlation was found between the Sorensen and SLS tests in Groups B (r = 0.02) and A (r = 0.16), and the TFET and OSI correlation was weakly positive in Group B (r = 0.15) but moderately negative in Group A (r = −0.46).

The core muscles primarily provide the main protection and stabilisation of the spine throughout dynamic and static phasic changes. They link our upper and lower body, allowing us to move freely or maintain a balanced standing position. A weak core can influence daily tasks such as walking, turning around, bending, dressing and showering. It will be difficult for the upper and lower limbs to move correctly if the core is weak or unstable. Core muscular endurance and balance are related since a balancing system disruption might impact daily activities [62,63]. The improvement of balance after core muscle training may be attributed to core muscles’ early stimulation by the central nervous system in anticipation of the motion of lower limbs. Due to this early activation, the lower limbs can move more freely as the base is more secure and stable [64].

The results of the current study are in accordance with the findings of the previous studies that found a positive relation between core muscle training and balance [64,65,66,67,68,69]. Kaji et al. [65] found that core stability exercises significantly reduce medial–lateral sway in healthy individuals. In addition, Barrio et al. [64] and Ozmen and Aydogmus [66] reported notable variations in core muscle endurance and dynamic balance in adolescents who underwent core training. Similarly, Ahmed et al. [67] and Dale et al. [68] reported that core muscle training enhances athletes’ dynamic balance and functional performance. Furthermore, Suri et al. [69] found that enhancement in the endurance of trunk extensors was correlated to alterations in dynamic and static balance in the elderly, suggesting that trunk extensors’ endurance has a more significant impact on dynamic than static balance.

On the contrary, De Los Ríos-Calonge et al. [70] reported no significant link between endurance and dynamic balance tests. The cause of this contradiction could be related to the different measurement conditions of trunk endurance concerning dynamic balance, as the body position assumed in the endurance tests of trunk muscles (sitting, bridging or quadruped positions) differs significantly from the standing position in the dynamic balance tests. Similarly, Ambegaonkar et al. [71] found no correlations between core endurance and balance. This might be due to the different tests used for balance assessment (Star Excursion Balance Test) and trunk endurance (McGill’s Core Endurance Test). The study was also conducted on athletes who might not have trunk endurance and balance impairments.

## 5. Limitations and Recommendations

Some limitations of the current study include the limited number of participants in the sample, the fact that the study focused only on young females, the low dose of training performed (only 4 min twice a week) and the lack of diet control. So, a large sample size can be used for future studies, and an assessment for different age groups and a comparison between males and females are recommended. A diet should be included in future studies for more improvement. The authors recommended that WBV is a potentially effective form of training. Further research should concentrate on assessing the long-term benefits of WBV on balance and trunk endurance in obese females.

## 6. Conclusions

In conclusion, the study’s parameters might help achieve neuromuscular alterations in trunk extensors, flexors and balance enhancement. Moreover, the suggested exercises can be easily carried out in everyday practice, making it a potentially beneficial training program for obese females to improve trunk endurance and balance, depending on a scientific basis.

## Figures and Tables

**Table 1:** WBV exercises.

Exercise	Description	Frequency	Duration
Light Squatting	From the standing position, the participant was instructed to bend the knees 10° with the feet turned out.	15 HZ	0–10 s
Standing in the erect position	The participant was asked to stand on the platform with the feet shoulder-width apart and the knee joint in full extension.	15 HZ	10–20 s
Standing in a relaxed position, with the knees in a slight flexion	The participant was instructed to assume a light squatting position by bending the knees 30°.	15 HZ	20–30 s
Light jumping	The participant was asked to gently lift the feet off the ground from standing on the platform and perform light jumping.	20 HZ	40–60 s
Alternating the body weight from one leg to another	The participant was instructed to stand on the platform and shift the body weight to the right lower limb, the left lower limb and so on.	25 HZ	40–60 s
Standing on the heels	From the standing position, the participant was asked to raise the toes off the ground and sustain this position.	30 HZ	50–60 s

**Table 2:** Demographic characteristics of the participants. Illustrates the characteristics of participants of Group A and Group B. No significant difference was reported in the mean age, height, weight or BMI (*p >* 0.05) between both groups.

Characteristics	Mean ± SD		MD	*p*	95% CI	
	WBV (*n* = 25)	Sham WBV (*n* = 25)			Lower	Upper
Age (years)	21.24 ± 2.03	20.96 ± 2.07	−0.28	0.63 (ns)	−1.45	0.89
Weight (Kg)	92.47 ± 10.51	93.96 ± 12.77	1.48	0.35 (ns)	−5.17	8.14
Height (cm)	158.3 ± 6.06	158.8 ± 5.94	0.52	0.76 (ns)	−2.89	3.93
BMI (kg/m^2^)	36.94 ± 4.99	37.62 ± 4.57	0.68	0.61 (ns)	−2.04	3.40

BMI: Body mass index, *n*: number, Mean ± SD is used to provide quantitative data, MD: Mean Difference, (ns): non-significant.

**Table 3:** Clinical measures.

Variable	WBV Group				Sham WBV Group				Group X Time Interaction (F) *p*-Value	*p* ^(b)^ between Groups	Effect Size (η^2^)
	Pre	Post	MD (95%CI)	*p* ^(a)^ within Group	Pre	Post	MD (95%CI)	*p* ^(a)^ within Group			
Sorensen test (s)	30.41 ± 17.46	48.41 ± 20.88	18.00 (14.58–21.41)	<0.001	28.08 ± 10.33	29.08 ± 10.22	1.00 (0.55–1.44)	0.65	F_1,48_ = 10.17	<0.001	0.33
									<0.01		
TFET (s)	10.08 ± 5.97	19.27 ± 10.54	9.19 (6.71–11.67)	<0.001	9.52 ± 2.47	10.00 ± 2.52	0.48 (0.26–0.69)	0.76	F_1.48_ = 16.10	<0.001	0.41
									<0.01		
SLS (s)	6.36 ± 2.92	18.05 ± 4.66	11.69 (10.11–13.28)	<0.001	6.30 ± 2.99	6.787 ± 3.05	0.48 (0.27–0.69)	0.78	F_1.48_ = 69.86	<0.001	0.71
									<0.001		
OSI (°)	3.51 ± 0.68	1.78 ± 0.0.59	−1.73 (−1.90 to −1.55)	<0.001	3.48 ± 0.76	3.12 ± 0.76	−0.36 (−0.42 to −0.29)	0.54	F_1.48_ = 80.20	<0.001	0.52
									<0.001		
MLSI (°)	2.63 ± 0.67	1.52 ± 0.63	−1.11 (−1.33 to −0.89)	<0.001	2.53 ± 0.63	2.38 ± 0.63	−0.15 (−0.19 to −0.10)	0.56	F_1.48_ = 25.57	<0.001	0.33
									<0.001		
APSI (°)	2.92 ± 0.77	1.44 ± 0.51	−1.48 (−1.72 to −1.24)	<0.001	2.86 ± 0.85	2.70 ± 0.84	−0.16 (−0.22 to −0.90)	0.63	F_1.48_ = 38.23	<0.001	0.51
									<0.001		

Values are shown as the mean ± standard deviation (SD); TFET: Trunk Flexor Endurance Test; SLS: Single-Leg Stance; OSI: Overall stability index; MLSI: Medial-lateral stability index.; APSI: Anterior–Posterior Stability Index; MD: Mean Difference; CI: Confidence Interval; *p* ^(a)^-value within the group from Paired-Samples T Test; *p* ^(b)^-value between WBV and Sham group post-intervention from Independent-Samples *t* Test; *p* < 0.05 is statistically significant; η^2^: partial eta square between WBV and Sham group post-intervention; Group X time interaction *p*-value from Two-way repeated measures ANOVA.

## Data Availability

The datasets used and/or analysed during the current study are available from the corresponding author upon reasonable request.
